# Based on network pharmacology and molecular docking to explore the molecular mechanism of Ginseng and Astragalus decoction against postmenopausal osteoporosis

**DOI:** 10.1097/MD.0000000000035887

**Published:** 2023-11-17

**Authors:** Wei Fan, Zong-Zhe Jiang, Sheng-Rong Wan

**Affiliations:** a Department of Orthopaedics, The Affiliated Hospital of Southwest Medical University, Luzhou, Sichuan, China; b Sichuan Provincial Laboratory of Orthopaedic Engineering, Luzhou, Sichuan, China; c Metabolic Vascular Disease Key Laboratory of Sichuan Province, Sichuan, China; d Department of Endocrinology and Metabolism, The Affiliated Hospital of Southwest Medical University, Luzhou, Sichuan, China; e Academician (Expert) Workstation of Sichuan Province, The Affiliated Hospital of Southwest Medical University, Luzhou, Sichuan, China; f Experimental Medicine Center, The Affiliated Hospital of Southwest Medical University, Luzhou, Sichuan, China.

**Keywords:** Ginseng and Astragalus decoction, molecular docking, network pharmacology, postmenopausal osteoporosis

## Abstract

Traditional Chinese medicine suggests that Ginseng and Astragalus Decoction (GAD) may effectively treat postmenopausal osteoporosis (PMO). However, the exact mechanism of action for GAD remains unclear. This study aims to utilize network pharmacology and molecular docking technology to explore the potential mechanism of GAD in treating PMO. The main chemical components of GAD were identified by consulting literature and traditional Chinese medicine systems pharmacology database. GeneCards and online mendelian inheritance in man were used to identify PMO disease targets, and Cytoscape 3.8.2 software was used to construct a herb-disease-gene-target network. The intersection of drug targets and disease targets was introduced into the search tool for the retrieval of interacting genes platform to construct a protein-protein interaction network. Additionally, we further conducted gene ontology and Kyoto encyclopedia of genes and genomes enrichment analyses, followed by molecular docking between active ingredients and core protein targets. We have identified 59 potential targets related to the treatment of PMO by GAD, along with 33 effective components. Quercetin and kaempferol are the compounds with higher degree. In the protein-protein interaction network, IL6, AKT1, and IL1B are proteins with high degree. The enrichment analysis of gene ontology and KEEG revealed that biological processes involved in treating PMO with GAD mainly include response to hormones, positive regulation of phosphorylation, and regulation of protein homodimerization activity. The signal pathways primarily include Pathways in cancer, PI3K-Akt signaling pathway, and AGE-RAGE signaling pathway. Molecular docking results indicate that kaempferol and quercetin have a high affinity for IL6, AKT1, and IL1B. Our research predicts that IL6, AKT1, and IL1B are highly likely to be potential targets for treating PMO with GAD. PI3K/AKT pathway and AGE-ARGE pathway may play an important role in PMO.

## 1. Introduction

Postmenopausal osteoporosis is a common metabolic bone disease in women.^[1,2]^ The pathogenesis of postmenopausal osteoporosis (PMO) remains unclear,^[1]^ but the mainstream view suggests that it is related to female physiological degeneration.^[3]^ Due to decreased estrogen secretion, postmenopausal women experience weakened inhibition of osteoclasts due to changes in immune status and the release of reactive oxygen species.^[4,5]^ This ultimately increases the risk of fracture, unnecessary bone pain, and other symptoms.^[6]^ Current treatment methods for PMO include raising estrogen levels,^[7]^ supplementing calcium,^[8]^ promoting calcium absorption,^[9]^ stimulating osteogenesis and inhibiting osteoclasts^[10]^; however, these drugs have limited efficacy in improving bone metabolism and may cause adverse reactions.^[11]^ Therefore, there is an urgent need to explore safer and more effective treatment strategies for PMO.^[12]^ Natural products are gaining attention due to their relative safety and potential anti-osteoporotic effects.^[13,14]^ Traditional Chinese medicine classifies PMO as “bone withered” and believes that “kidney and vital essence deficiency” are the main pathogeneses of PMO. Kidneys govern bones by generating marrow; kidney deficiency leads to passive vital essence depletion which results in increased bone fragility leading ultimately to PMO.

Ginseng is a highly valued medicinal material in traditional Chinese medicine due to its ability to tonify the kidney and replenish vital essence. Studies have shown that ginseng extract can improve symptoms of osteoporosis and arthritis in women.^[15]^ Although traditional Chinese medicine has long used ginseng for postmenopausal osteoporosis treatment, it lacks clinical analysis and experimental demonstration. Astragalus (AS) is a leguminous plant known for its direct role in tonifying the kidney according to ancient Chinese medicine books. Rihuazi Materia Medica records AS as being able to “invigorate qi and strengthen muscles and bones.” Clinical studies have demonstrated that AS can increase lumbar bone density, inhibit bone absorption, maintain positive balance by adjusting bone metabolism, and prevent bone loss.^[16,17]^ Traditional Chinese medicine has a history of using AS for treating osteoporosis; however, research on its mechanism remains deficient.

Traditional Chinese Medicine has long used GAD to treat PMO. The combination of Ginseng and AS usually has a synergistic effect. However, this method only exists in ancient Chinese medical books and passed down experiences, lacking mechanism research. Due to the complexity of traditional Chinese medicine components, multiple targets, and broad signaling pathways, in-depth research is challenging. Network pharmacology is an emerging analytical strategy that uses virtual computing and database retrieval. It employs techniques like high-throughput screening, network visualization, and analysis to uncover complex biological network relationships involving drugs, genes, targets, and diseases. This helps predict drug mechanisms and provides references for understanding drug effects.^[18]^ The goal of network pharmacology research is to systematically address scientific challenges at multiple levels. Interestingly, this theory aligns with the concept of preventive treatment in traditional Chinese medicine known as syndrome differentiation.^[19]^ By analyzing existing databases, network pharmacology can study traditional Chinese medicines and diseases. It offers a novel approach to overcome limitations in basic research on traditional Chinese medicine.^[20]^

Based on this situation, we established an herb-disease-gene-target network. Then we obtained the main signaling pathways and biological functions through gene ontology (GO) analysis and Kyoto encyclopedia of gene and genome (KEGG) enrichment analysis. Finally, molecular docking technology was used to verify the proposed functional components and major targets. This study screened active ingredients with practical value in GAD through network pharmacology, determined their target roles and interactions while predicting the therapeutic mechanism of GAD against PMO. This provides useful reference for future research in this field.

## 2. Materials and methods

Ethical approval was waived or not necessary, all procedures performed in studies do not involve human participants or animals.

### 2.1. Screening the active ingredients of GAD

We utilized the traditional Chinese medicine systems pharmacology database (TCMSP) platform (http://tcmspw.com/tcmsp.php, accessed in March, 2023) to identify the active components of GAD. Our selection criteria were based on absorption, distribution, metabolism and excretion processes. Specifically, we screened for ingredients with oral bioavailability ≥30% and drug-like properties ≥0.18 in order to obtain appropriate active compounds.^[21,22]^

### 2.2. Identification of PMO prediction targets and construction of herb-disease-gene-target network

After identifying the effective target of GAD in the TCMSP database, we utilized the Uniprot database (https://www.uniprot.org/, accessed in March, 2023) to standardize target and gene symbols. To obtain disease targets, we entered the keyword “postmenopausal osteoporosis” into both online mendelian inheritance in man (https://omim.org/, accessed in March, 2023) and Genecards (https://www.genecards.org/, accessed in March, 2023) databases.^[23,24]^ We then merged the targets from both databases, removed duplicates, and collected the remaining targets as PMO targets. Using Cytoscape 3.8.2 network visualization software, we constructed a network of effective components and action targets for “herb - disease - gene - target.” The network analyzer plug-in was used to analyze network characteristics and clarify interactions between effective components and targets.

### 2.3. Protein-protein interaction (PPI) network construction and analysis

The search tool for the retrieval of interacting genes/proteins (STRING) (https://string-db.org/, accessed in March, 2023) was used to predict the interaction between proteins. The protein-protein interaction network (PPI network) was created by introducing overlapping targets.^[25]^ Cytoscape was used to construct the core PPI network.

### 2.4. GO enrichment and KEGG pathway analysis

To elucidate the biological functions and signaling pathways associated with PMO, we conducted GO and KEGG enrichment analysis using the Metascape database (https://metascape.org/, accessed in March, 2023).^[26]^ The GO analysis identified relevant biological processes, cellular components, and molecular functions (MF). Additionally, KEGG enrichment analysis enabled us to identify significant signaling pathways involved in these biological processes. The *P* value cutoff was set at *P* < .01, indicating that a lower *P* value suggests a higher probability of the current result being a genuine enrichment outcome rather than a random event. In this study, we used −log 10 (*P* value) as the unit, and as this value increased, the reliability of the enrichment results also increased.

### 2.5. Molecular docking

Molecular docking is a theoretical simulation method used in drug design to predict the binding modes and affinities of molecules by studying their interactions with receptors and ligands.^[27]^ In this study, we employed molecular docking to investigate whether the core components of GAD identified through network pharmacology could bind with core proteins. We selected the top 2 compounds based on degree value from the “herb-disease-gene-target” network core components of GAD, and chose the top 3 proteins based on degree value from PPI network core targets. Corresponding 3D structure files for proteins and small molecule compounds were downloaded from RCSB database (https://www.rcsb.org/, accessed in March, 2023) and TCMSP database^[28]^, followed by dehydration and hydrogenation before importing them into Autodock Tools (ver.1.5.6). The small molecule compound was then docked with protein as receptor using molecular docking method, selecting the most suitable conformation. Finally, we generated a 3D binding mode diagram using Pymol (ver.1.8.x).^[29]^

## 3. Results

### 3.1. Acquisition of natural components of GAD

According to the screening criteria, oral bioavailability ≥30% and drug-likeness ≥0.18 were set in the TCMSP database for screening. The results showed that a total of 33 active components of GAD complex were obtained, and the details were shown in table 1.

**Table 1:** Effective compounds of Ginseng and Astragalus decoction.

Name	Mol ID	Mol name	OB (%)	DL
GS1	MOL002879	Diop	43.59	0.39
GS2	MOL000449	Stigmasterol	43.83	0.76
GS3	MOL000358	Beta-sitosterol	36.91	0.75
GS4	MOL003648	Inermin	65.83	0.54
GS5	MOL005308	Aposiopolamine	66.65	0.22
GS6	MOL005317	Deoxyharringtonine	39.27	0.81
GS7	MOL005318	Dianthramine	40.45	0.2
GS8	MOL005320	Arachidonate	45.57	0.2
GS9	MOL005321	Frutinone A	65.9	0.34
GS10	MOL005344	Ginsenoside rh2	36.32	0.56
GS11	MOL005348	Ginsenoside-Rh4_qt	31.11	0.78
GS12	MOL005356	Girinimbin	61.22	0.31
GS13	MOL005376	Panaxadiol	33.09	0.79
GS14	MOL005384	Suchilactone	57.52	0.56
GS15	MOL005399	Alexandrin_qt	36.91	0.75
GS16	MOL000787	Fumarine	59.26	0.83
AS1	MOL000211	Mairin	55.38	0.78
AS2	MOL000239	Jaranol	50.83	0.29
AS3	MOL000295	Alexandrin	20.63	0.63
AS4	MOL000296	Hederagenin	36.91	0.75
AS5	MOL000033	(3S,8S,9S,10R,13R,14S,17R)-10,13-dimethyl-17-[(2R,5S)-5-propan-2-yloctan-2-yl]-2,3,4,7,8,9,11,12,14,15,16,17-dodecahydro-1H-cyclopenta[a]phenanthren-3-ol	36.23	0.78
AS6	MOL000354	isorHamnetin	49.6	0.31
AS7	MOL000371	3,9-di-O-methylnissolin	53.74	0.48
AS8	MOL000378	7-O-methylisomucronulatol	74.69	0.3
AS9	MOL000379	9,10-dimethoxypterocarpan-3-O-β-D-glucoside	36.74	0.92
AS10	MOL000380	(6aR,11aR)-9,10-dimethoxy-6a,11a-dihydro-6H-benzofurano[3,2-c] chromen-3-ol	64.26	0.42
AS11	MOL000387	Bifendate	31.1	0.67
AS12	MOL000392	Formononetin	69.67	0.21
AS13	MOL000417	Calycosin	47.75	0.24
AS14	MOL000433	FA	68.96	0.71
AS15	MOL000442	1,7-Dihydroxy-3,9-dimethoxy pterocarpene	39.5	0.48
AS16	MOL000098	Quercetin	46.43	0.28
A1	MOL000422	Kaempferol	41.88	0.24

The common component of Ginseng and Astragalus.

A1 = kaempferol, AS = Astragalus, DL = drug-likeness, GS = Ginseng, Mol = molecular, OB = oral bioavailability.

### 3.2. Identification of core targets and construction of herb-disease-gene-target network

**Figure 1.**
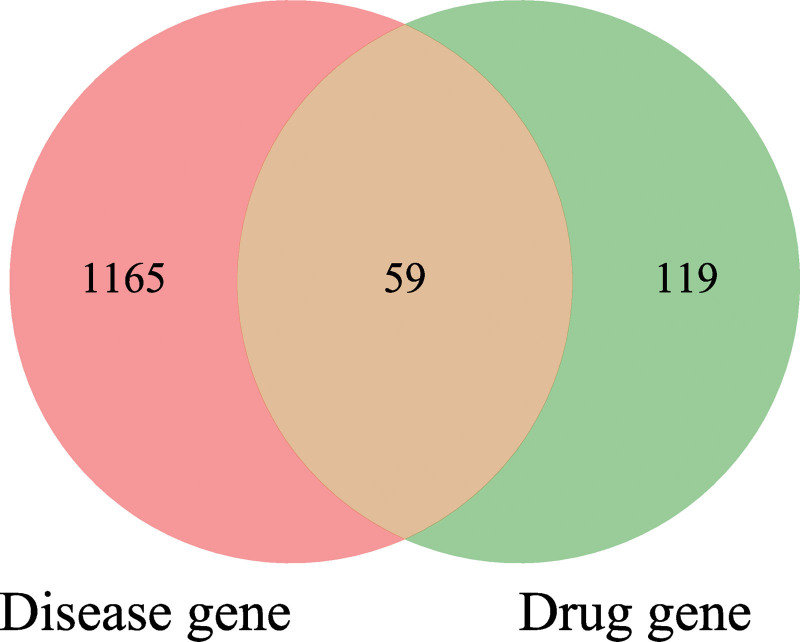
Venn diagram showing the overlapping target genes for GAD against PMO. GAD = Ginseng and Astragalus decoction, PMO = postmenopausal osteoporosis.

**Figure 2.**
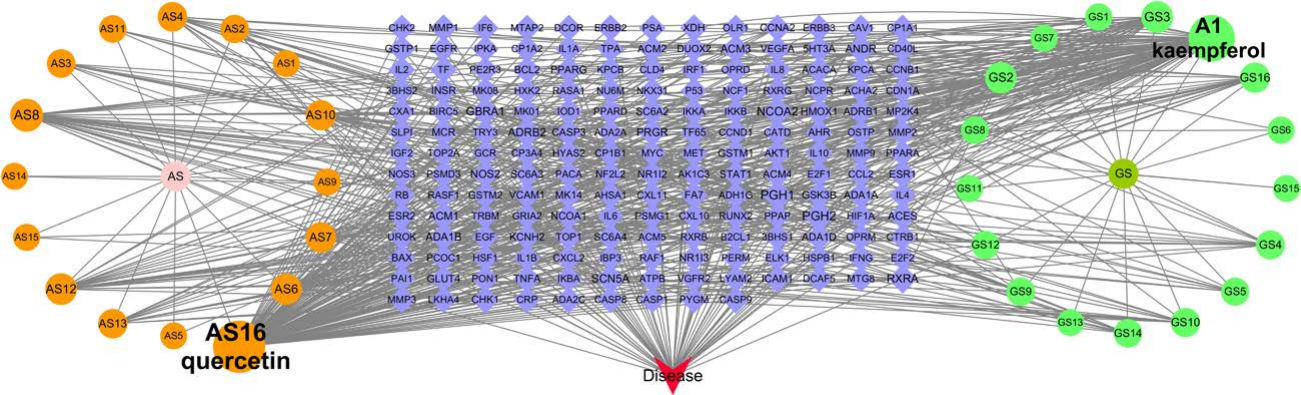
Herb - disease - gene - target network of GAD against PMO. The light purple diamond represents overlapping target genes. The yellow and green circulars represent the active components of AS and GS respectively, and A1 represents the common components of AS and GS. The larger the font size, the more important its role in the compound. AS = Astragalus, GS = Ginseng, PMO = postmenopausal osteoporosis, A1 = the common components of AS and GS.

We conducted a screening of PMO disease-related targets in online mendelian inheritance in man and GeneCards databases, following standard procedures and removing repeated targets. This yielded 1224 potential targets. We then sorted the predicted results from TCMSP and literature review, removed duplicates, matched them with corresponding disease targets, resulting in 59 core targets. A Venn diagram (Fig. 1) was drawn to illustrate this process. To further observe compound-target interactions, we constructed an herb-disease-gene-target network (Fig. 2). Our findings indicated that 2 compounds - quercetin (AS16; degree = 124) and kaempferol (A1; degree = 96) - were highly correlated with PMO targets.

### 3.3. Construction and analysis of PPI network

**Figure 3.**
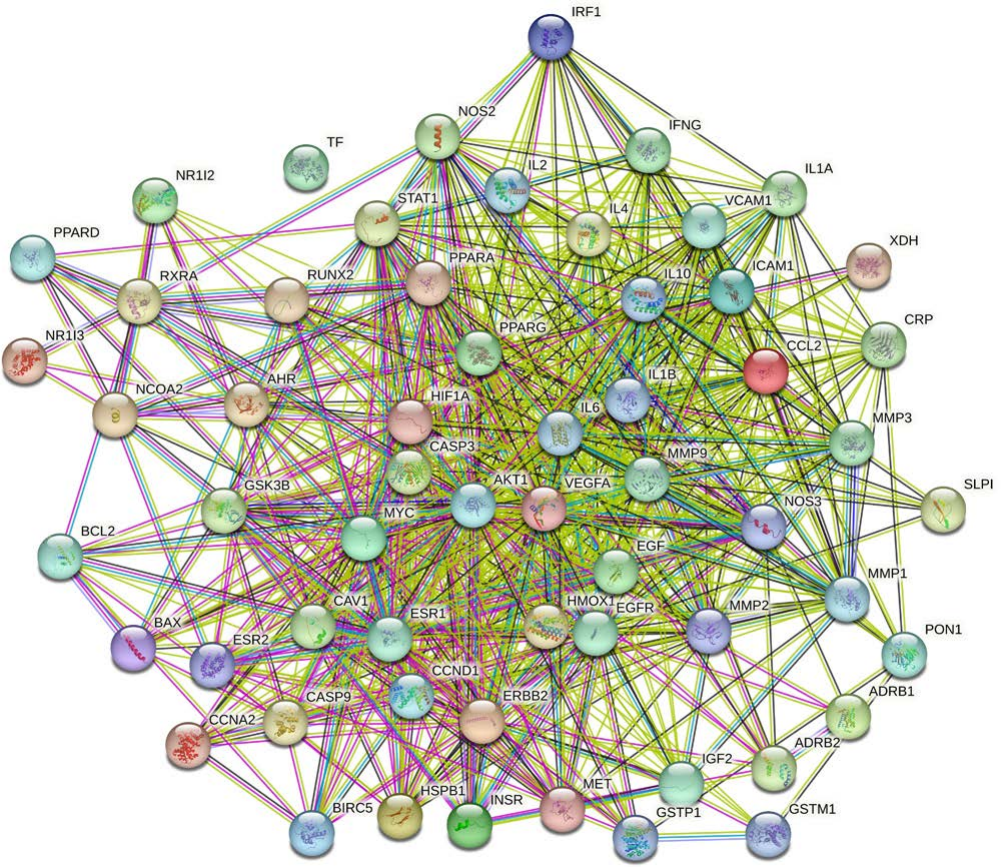
PPI network. Empty nodes represent proteins of unknown 3D structure; filled nodes represent some 3D structure that is known or predicted. Edges represent protein–protein associations: the light blue edges represent from curated databases; the fuchsia edges represent experimentally determined; the green edges represent gene neighborhood; the red edges represent gene fusions; the dark blue edges represent gene co-occurrence; the light green edges represent text mining; the black edges represent co-expression; the light purple edges represent protein homology.

**Figure 4.**
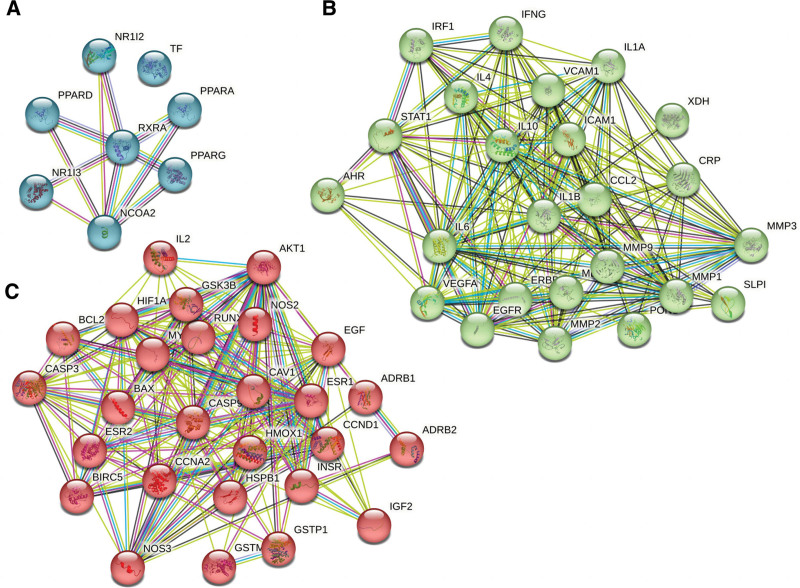
The central gene cluster identified in the PPI network of GAD-PMO based on MCODE analysis. (A–C was cluster 1, 2, and 3, respectively). Colored lines represent the evidence which supports the interaction between different target proteins and the colored balls represent the different target proteins. PMO = postmenopausal osteoporosis.

**Figure 5.**
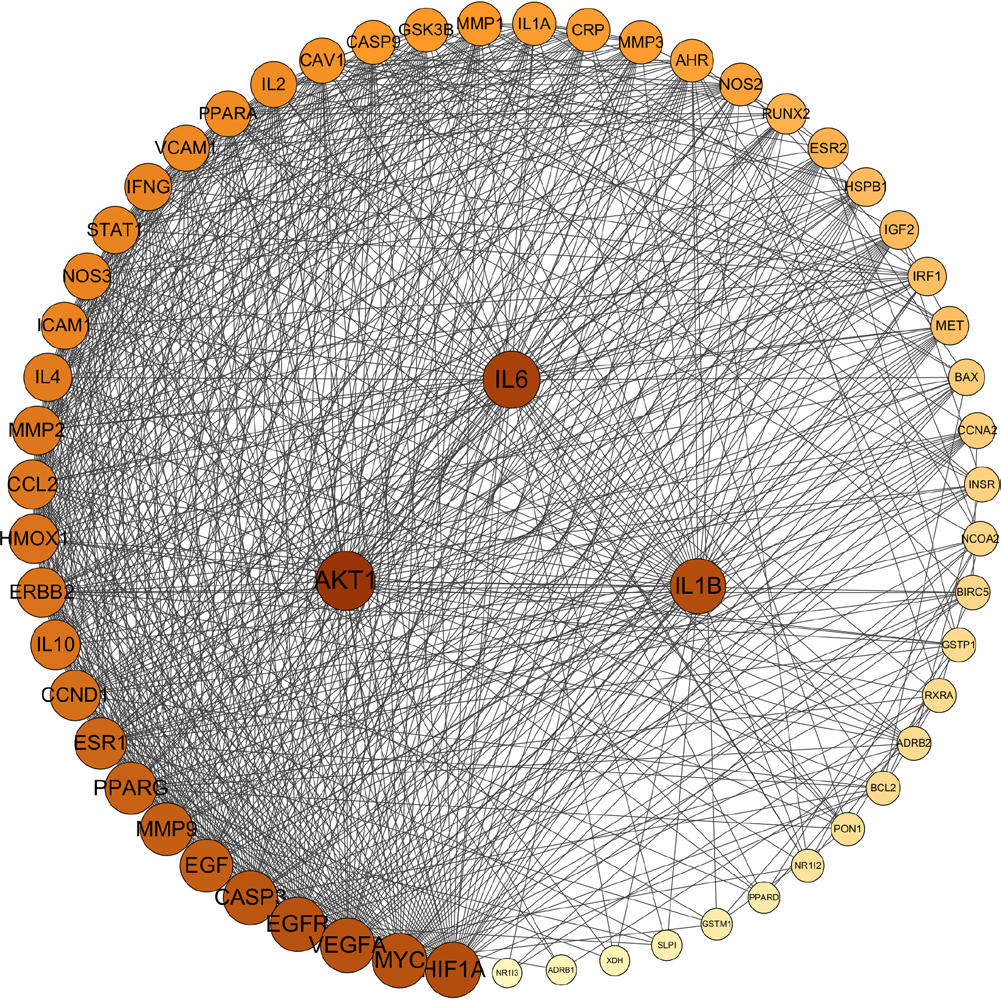
PPI network diagram processed by Cytoscape. The color of the target point changes gradually according to the degree value. The higher the degree value, the larger the circle. The color changes from light yellow to dark yellow with the change of degree value.

We imported the core targets of disease and drug components into the STRING database to display the PPI network diagram (Fig. 3). Molecular complex detection (MCODE) is a novel graph-theoretic clustering algorithm that calculates the information of each node in a PPI network graph using a k-means clustering algorithm to detect dense connection regions in large protein-protein interactions. Proteins within these regions may have similar structures or functions, providing a reference and guidance for further research on protein-disease interactions.^[30]^ We further analyzed the target information of the PPI network queried from the STRING database by MCODE, which showed 3 central gene clusters. This indicates that proteins in these clusters are closely connected and may have common functions or expression patterns (Fig. 4). To identify key target proteins, we used Cytoscape software (Fig. 5). The degree of a protein reflects its frequency of interaction, strength of interaction between targets, and importance in the PPI network. Therefore, a higher degree suggests that it may be a central target for potential treatment with GAD active ingredient for PMO. For instance, AKT1 (degree = 50), IL6 (degree = 47), and IL1B (degree = 44) are such key targets.

### 3.4. GO enrichment and KEGG pathway analysis

**Figure 6.**
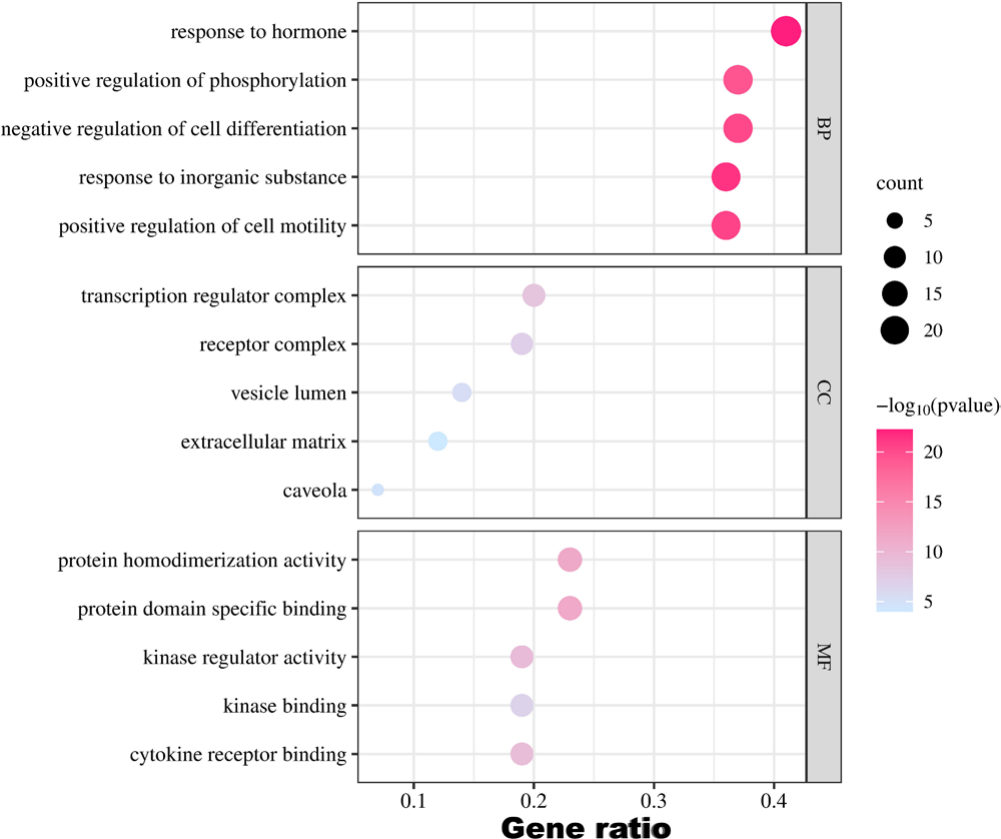
GO analysis of key target genes. The top 6 items of biological function are listed on the vertical axis, including GOMF, GOCC, and GOBP, the horizontal axis in the figure represents the gene ratio. GO = gene ontology, BP = biological process, CC = cell composition, MF = molecular function.

**Figure 7.**
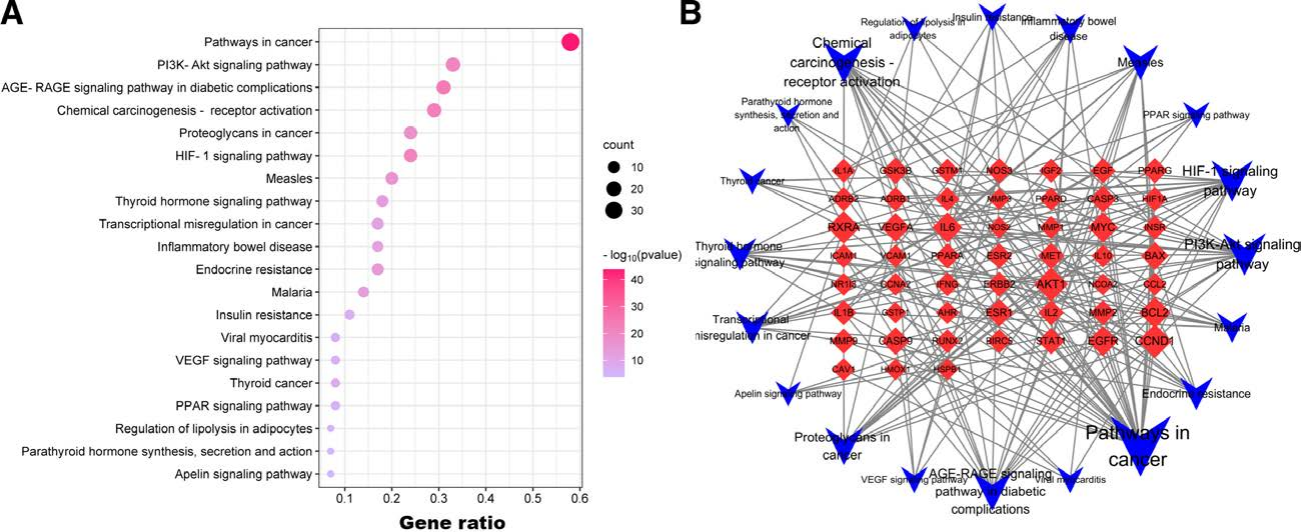
(A) KEGG analysis of key target genes, (B) Network of top 20 pathways. Red diamond represents gene, and blue triangle represents pathway. The size of the nodes represents the value of the degree. The horizontal axis in the figure represents the gene ratio. KEGG = Kyoto encyclopedia of gene and genome.

To clarify the possible pathway and biological function of GAD in treating PMO, we conducted GOBP, GOCC, GOMF analysis, and KEEG enrichment analysis. Figure 6 displays the first 5 results of our GO analysis. The results suggest that GAD treatment for PMO is likely related to hormone response. Additionally, our analyses indicate that transcription regulator complex is the most important cell component while regulating protein homodimerization activity is the most likely MF (as suggested by GOCC and GOMF). Our KEGG analysis results are shown in Figure 7 and include Pathways in cancer, PI3K-Akt signaling pathway, AGE-RAGE signaling pathway in diabetic complications.

### 3.5. Molecular docking

**Figure 8.**
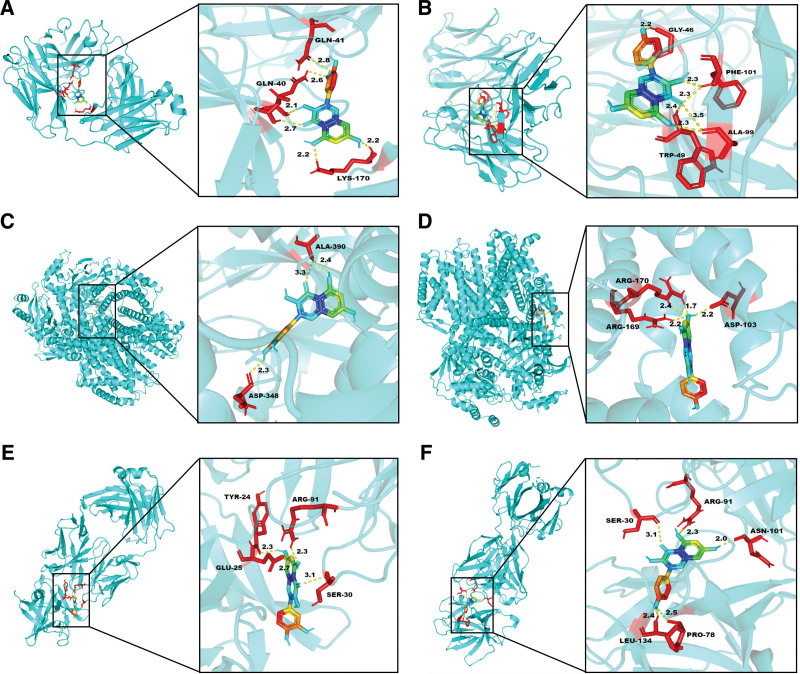
Molecular docking results of main chemical components of GAD and core proteins in PPI network (A) IL6-quercetin; (B) IL6-kaempferol; (C) AKT1-quercetin; (D) AKT1-kaempferol; (E) IL1B-quercetin; (F) ILIB-kaempferol.

We employed network pharmacology and protein-protein interaction (PPI) analysis to determine the top 2 compounds, namely quercetin and kaempferol, for docking with the top 3 proteins IL6, AKT1, and IL1B. The best docking image of the receptor-ligand complex is presented in Figure 8.

The results indicate that quercetin and kaempferol can effectively interact with IL6, AKT1, and IL1B. Specifically, IL6 forms hydrogen bonds with quercetin through GLN-41, GLN-40, and LYS-170 (Fig. 8A), while it interacts with kaempferol via GLY-46, PHE-101, TRP-49, and ALA-99 (Fig. 8B). Similarly, AKT1 forms hydrogen bond connections with quercetin through ALA-390 and ASP-348 (Fig. 8C) and interacts with kaempferol via ARG-170, ARG-169, and ASP-103 (Fig. 8D). Finally, IL1B forms hydrogen bond connections with quercetin through TYR-24, GLU-25, ARG-91, and SER-30 (Fig. 8E), whereas it interacts with kaempferol via SER-30, ARG-91, ASN-101, LEU-134, and PRO-78 (Fig. 8F).

The affinity between the receptor and ligand increases as the binding energy decreases, resulting in a more stable conformation. A binding energy of <−5 kcal/mol is generally considered indicative of good binding activity and affinity.^[31,32]^ Our molecular docking results reveal that the compounds we screened have high affinity with the core target, as their binding energies are all <−5 kcal/mol. The specific docking energies can be found in Table 2. Quercetin and kaempferol, exhibit strong bonding with 3 core targets (IL6, AKT1, and IL1B). Therefore, we predict that these compounds may play a crucial role in treating PMO.

**Table 2:**
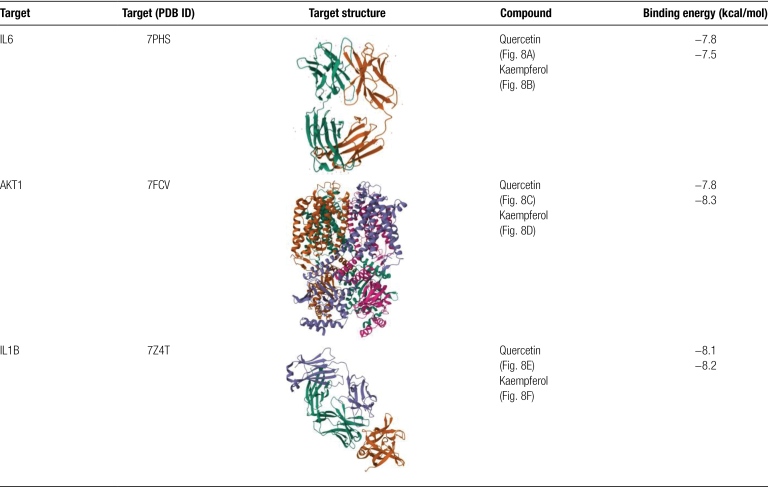
Binding energy of molecular docking.

## 4. Discussion

PMO is a metabolic disease that women are prone to suffer from after menopause. Once it is not treated in time, it may lead to fracture, which seriously threatens the life and health of postmenopausal women. The traditional Chinese medicine GAD has been used in the treatment of PMO for a long time, but its mechanism and target are still unclear. The main purpose of this research is to explore the potential mechanism of GAD in the treatment of PMO. Through the analysis of traditional Chinese medicine compound database and disease database and the construction of network diagram, we speculate that the main chemical components of GAD in the treatment of DF include quercetin and kaempferol etc. Animal research shows that quercetin rescued TNF-alpha-induced impairments in bone marrow-derived mesenchymal stem cell osteogenesis and improved osteoporosis in rats.^[33]^ Other studies have shown that quercetin alleviate ovariectomy-induced osteoporosis by modulating autophagy and apoptosis in rat bone cells.^[34]^ However, the current in vitro research mainly focuses on the prevention and potential mechanism of quercetin on PMO.^[35]^ For the first time, we demonstrated the role of quercetin in PMO through the mining of drug components, construction of network targets and molecular docking verification, which well corroborated previous views and provided a good reference for the next in vivo research or clinical trials of quercetin in the treatment of PMO. With regard to the effect of kaempferol on PMO, only studies have shown that kaempferol has an inhibitory effect on bone loss in rats with osteoporosis after ovariectomy,^[36]^ but there is still no study on its potential mechanism and clinical effect. Our research is the first to predict the mechanism of action of kaempferol in the treatment of PMO through network pharmacology and drug composition mining, which provides a strong reference basis for subsequent research, which is our significance.

Through the construction of PPI network, we can find that IL6, AKT1, and IL1B are the core proteins of GAD in treating PMO. Some studies have shown that IL-6 is an important pathogenic factor of immune-mediated bone diseases such as rheumatoid arthritis and postmenopausal osteoporosis. IL-6 mediates the actions of osteoblasts and osteoclasts through sophisticated mechanisms, which reflect dual effects.^[37]^ IL1B is a member of the IL-1 family. It has been proved that IL1B can regulate the chronic inflammatory response of many diseases, such as Parkinson disease,^[38]^ amyotrophic lateral sclerosis^[39]^ idiopathic tremor^[40]^ Alzheimer disease.^[41]^ Research on the polymorphism of IL-1B has been carried out worldwide.^[42]^ At present, both in vitro and in vivo experiments also show that IL-1B can significantly affect cartilage destruction and bone resorption.^[43–45]^ However, the research on the effect of IL1B on PMO is still rare, which may also be a future research direction.

KEGG analysis enrichment analysis results mainly include pathway in cancer, PI3K-Akt signaling pathway and AGE-RAGE signaling pathway. Previous studies have shown that quercetin, in addition to reducing osteoporosis by regulating autophagy and apoptosis of bone cells, also participates in many molecular mechanisms of cancer occurrence, which may be the main reason for the enrichment of pathways in cancer.^[46–48]^ Recent studies have shown that PI3K/AKT pathway plays an important role in the process of cell apoptosis.^[49,50]^ In addition, PI3K/AKT pathway is closely related to cell proliferation and apoptosis.^[51,52]^ Furthermore, AKT and PI3K are generally downregulated when osteoporosis occurs, illustrating that the progression of osteoporosis might be associated with cell injury.^[35,53,54]^ There are many other studies on the role of PI3K/AKT pathway in PMO, for example, silence of miR-483-5p can alleviate postmenopausal osteoporosis through PI3K/AKT pathway^[55]^; Chlorogenic acid can prevent osteoporosis in ovariectomized rats through PI3K/Akt pathway.^[56]^ Therefore, PI3K/AKT pathway is an important pathway for the occurrence and development mechanism of PMO, and also an important medium for the treatment of PMO. Our research speculates that GAD treatment of PMO is probably achieved through PI3K/AKT pathway. This also confirms the prediction result of our PPI network, that is, AKT1 plays an important role in the treatment of PMO with GAD.

AGE-RAGE signaling pathway has been considered as an important signaling pathway for complications of diabetes,^[57–59]^ but the latest systematic review shows that AGE-RAGE signaling inhibitors are the latest therapeutic targets for age-related diseases such as osteoporosis, rheumatoid arthritis, etc.^[60]^ Another animal study showed that lycopene can regulate bone metabolism through AGE/RAGE pathway, thus delaying the progress of osteoporosis in mice.^[61]^ In addition, diabetes itself can damage the metabolism and function of bone cells, leading to an increased risk of brittle fracture, which may also be the embodiment of AGE-RAGE signaling pathway in PMO. Therefore, combined with our research, we believe that the research on AGE-RAGE signaling pathway should not be limited to the complications of diabetes, it may have effects on a variety of metabolic diseases, which may also be a future research direction.

GOBP results suggest that the treatment of PMO by GAD is most likely related to the response to hormone. The analysis of GOMF and GOCC respectively suggested that the most important cell component was transcription regulator complex, and the most likely MF is the regulation of the activity of protein homodimerization. Since postmenopausal osteoporosis itself involves a series of hormone imbalance processes,^[62,63]^ we are not surprised by the results of GOBP. The research results of GOMF and GOCC also revealed some MFs of GAD in treating PMO, and also provided some reference for the pathogenesis of PMO.

Based on the above discussion, we can boldly guess that small molecules quercetin, kaempferol and protein IL6, AKT1, IL1B play an important role in the treatment of PMO by GAD. Therefore, we conducted molecular docking between the screening results of compound active ingredients and the main targets in PPI network to verify the predicted results of our network pharmacology. The docking results showed that the 3 compounds could bind well with protein, and their minimum binding energy was <−5 kJ/mol. Thankfully, the molecular docking results have well verified our prediction. It is precisely because the therapeutic effects and mechanisms of these potential active ingredients on PMO have not been explained and verified, so we believe that the development space and significance are great.

This study still has some limitations. We used modern bioinformatics methods to explore the role of GAD in PMO through network pharmacology and molecular docking. However, at present, the network information technology needs to be further improved, and the accuracy and timeliness of database data need to be scientifically verified. Meanwhile, although quercetin and kaempferol are considered to be the most important bioactive components for treating GAD in PMO, they cannot fully represent the GAD. Therefore, pharmacodynamic and molecular biological experiments need to be considered to further verify our research results. Additionally, clinical studies are still required to determine the effectiveness of GAD in treating PMO. This is the focus of our next work.

## 5. Conclusions

In this study, we first used the method of combining network pharmacology and molecular docking to clarify the mechanism of GAD in treating PMO. Quercetin and kaempferol may be the main effective compounds for treating PMO through GAD therapy. They may slow down the progress of osteoporosis by regulating cell autophagy, inhibiting bone loss, and inducing osteogenesis. In addition, we proposed that PI3K/AKT pathway and AGE/ARGE pathway may play an important role in postmenopausal osteoporosis and predicted the future research direction. In conclusion, this study provides theoretical support for further in-depth study of the mechanism of GAD in treating PMO, and provides possible future research directions and reference basis for the pathogenesis of PMO and new treatment methods. However, further clinical experiments and in-depth basic research are necessary to fully comprehend the therapeutic effects and molecular mechanisms of traditional Chinese medicine on PMO.

## Acknowledgment

The authors would like to thank all authors of references.

## Author contributions

**Conceptualization:** Wei Fan, Sheng-Rong Wan.

**Data curation:** Wei Fan, Sheng-Rong Wan.

**Formal analysis:** Wei Fan, Sheng-Rong Wan.

**Investigation:** Zong-Zhe Jiang.

**Methodology:** Zong-Zhe Jiang.

**Project administration:** Zong-Zhe Jiang.

**Resources:** Zong-Zhe Jiang.

**Software:** Wei Fan, Zong-Zhe Jiang.

**Supervision:** Sheng-Rong Wan.

**Validation:** Sheng-Rong Wan.

**Visualization:** Sheng-Rong Wan.

**Writing – original draft:** Wei Fan, Zong-Zhe Jiang, Sheng-Rong Wan.

**Writing – review & editing:** Wei Fan, Zong-Zhe Jiang, Sheng-Rong Wan.
